# Challenges and barriers of humanitarian aid management in 2017 Kermanshah earthquake: a qualitative study

**DOI:** 10.1186/s12889-020-08722-5

**Published:** 2020-04-25

**Authors:** Hamid Safarpour, Saeideh Fooladlou, Meysam Safi-Keykaleh, Somayyeh Mousavipour, Davoud Pirani, Ali Sahebi, Hassan Ghodsi, Iman Farahi-Ashtiani, Arezoo Dehghani

**Affiliations:** 1 Department of Nursing, Faculty of Nursing and Midwifery, Ilam University of Medical Sciences, Ilam, Iran; 2 Department of Health in Disasters and Emergencies, School of Public Health and Safety, Shahid Beheshti University of Medical Sciences, Tehran, Iran; 3 Department of Healthcare Services Management, School of Management and Medical Informatics, Tabriz University of Medical Sciences, Tabriz, Iran; 4 Malayer School of Nursing, Hamadan University of Medical Sciences, Hamadan, Iran; 5 Department of Nursing, School of Nursing and Midwifery, Neyshabur University of Medical Sciences, Neyshabur, Iran

**Keywords:** Disaster, Donation management, Humanitarian aid, Aid distribution, Iran

## Abstract

**Background:**

Examining various problems after disasters is important for the affected people. Managing humanitarian aid and donations among the affected people is considered as one of the most important problems after disasters. Therefore, the present study aimed to evaluate the challenges and barriers of humanitarian aid management in 2017 Kermanshah Earthquake.

**Methods:**

This study was conducted from November 2017 to January 2018, with qualitative case study design. The population included 21 people including 6 humanitarian aid manager, 6 volunteers, 4 aid workers, and 5 affected people. The data were collected through semi-structured interviews and purposeful sampling, which continued until saturating the data. The strategies recommended by Guba were used for evaluating the trustworthiness of the data. The data was analyzed with conventional content analysis method according to method suggested by Graneheim and Lundman.

**Results:**

Based on the results, two themes, nine categories, and 19 sub-categories were identified considering the challenges and barriers of humanitarian aid and donors’ management during the 2017 Kermanshah Earthquake. The categories included education, command and coordination, communication and information, rules, security, traffic and overcrowding, assessment, providing system, and cultural setting. Also, two themes including managerial and structural barriers were extracted.

**Conclusion:**

Adopting an effective management and appropriate policies with respect to humanitarian aid and modifying structural and managerial barriers can improve the performance and management of humanitarian aid.

## Background

Community involvement and collaboration in all of the phases in disaster management are essential for achieving a successful disaster management. However, the type of assistance can vary according to the characteristics of each country [1]. In most cases, national and international aid agencies provide technical and financial assistance to the victims after a disaster [2]. UNDRO (1982) introduced the community participation approach as a key success in the post-disaster reconstruction [3]. After disasters, humanitarian organizations mobilize a large amount of aid to disaster area. However, the distribution of these resources creates many challenges such as time, cost, coverage, and eligibility [4]. Extreme disasters like storms, earthquakes, tsunamis, and volcanic eruptions often bring extraordinary acts of helping [5]. During the humanitarian aid, the donors want to spend their money on alleviating the immediate distress [6]. The competition of aid agencies for donor resources can be considered as a barrier for collaboration and cooperation [7]. Humanitarian aid distribution is related to many activities such as involving a large number of stakeholders for managing disaster, enormous effort, and different processes. Humanitarian aid distribution is associated with many activities such as accessing up-to-date and appropriate information for effective communication in order to prompt collaboration and improve integration [8]. Regarding disaster management, several stakeholders are involved in managing the distribution of humanitarian aid. The stakeholders such as Governmental Organizations (GOs), Nongovernmental Organizations (NGOs), volunteers, and private sectors are responsible for managing humanitarian aid for disaster victims [9]. In addition, collaboration and coordination are considered as the most important factors in managing donations during disasters [10]. However, humanitarian aid in different situations is always accompanied by many challenges. Due to the lack of political engagement, manipulation by state authorities can result in perverting aid interventions [11].

A powerful earthquake struck Kermanshah province in west of Iran near the Iran-Iraq border at 18:18:16 UTC (21,48:16 local time) on November 12, 2017. More than 600 people lost their lives, about 10,000 were injured, and thousands of building were damaged [2, 12]. Consequently, thousands of the affected people were displaced in Sar-e Pul-e Zahab, Ezgeleh and surrounding villages [2], which needed urgent government and humanitarian assistance, especially in the early days of the disaster. However, humanitarian aid began by GOs, NGOs, and public community immediately after the earthquake. However, some problems and challenges were created for managing humanitarian aid. Thus, the present study aimed to evaluate the challenges and barriers for humanitarian aid management in 2017 Kermanshah earthquake.

## Methods

### Study design

A qualitative case study design was applied for conducting the study. Case studies designs are in-depth examinations of people, groups of people, or institutions [13]. First, the data were collected directly and without any pre-suppositions. Then, the codes, sub-categories, categories, and themes were derived through an inductive process. In fact, qualitative content analysis is a research method used to interpret the content of text data. In addition, the conventional content analysis is generally used in designing the study aiming to describe a phenomenon. This design is often appropriate when the theory or the research literature about a phenomenon is limited [14]. The researchers avoid using the pre-assumed notions, while they allow the categories and codes to be extracted from the data. In this study, the codes, sub-categories, categories, and themes were extracted by an inductive process and were conceptually ordered by considering developed properties and dimensions [15].

### Participants

This study was conducted in Kermanshah province, Iran from November 2017 to January 2018. A total of 21 participants were selected for interviews in terms of demographic characteristics. The participants were selected based on purposeful sampling employing maximum variety sampling. The inclusion criteria included the life experience of the current earthquake, either as affected people, manager, or key informant, and the willingness to participate in the interviews. However, those individuals who refused to give informed consent were eliminated. The participants included six humanitarian aid managers, six volunteers, four aid workers, and five affected people. The questions for a semi-structured interview were designed based on a deep interview with the interviewees and the texts were transcribed. The interview questions were reviewed and approved by the researchers several times. After each interview, the researchers listened to the recorded audio file several times and transcribed them as soon as possible. In addition, taking notes, along with recording the data during the interview was considered. Interviews reached saturation in the 21 interview and data collection was stopped at this stage.

### Data collection and analysis

Data were collected through in-depth and semi-structured interviews, and field observation. Since researchers seek for the meaning of individuals' experiences, asking individuals and understand their experiences through interviews is considered as the best strategy for obtaining the meaning of experiences. The participants were requested to read and sign an informed consent form and invited to participate. Initially, the first two in-depth interviews were performed following by semi-structured interview to explore the participants' experiences. The semi-structured interviews were conducted among well-experienced and/or knowledgeable key person including humanitarian aid mangers, aid workers, and help volunteers. The first question was related to the participants' experiences. The main points of questions which only served as an interview guide were *'Please describe your experience of the earthquake?"*, “*How about humanitarians and aids and donation?”*, and *“What are the barriers to aid and donations management?”*. Then, these questions continued with in-depth questions such as “*Could you please explain more?*”, and “*Take a sample from your own experience*”. After establishing the basic concepts, the interview questions were designed based on the gaps in the previous interviews. Thus, the questions were different in various interviews. The interview continued to saturate the concepts. Each interview lasted between 30 and 60 min. All interviews were recorded, transcribed verbatim, and analyzed using constant comparative method. The interviews were conducted in Persian and then translated into English by a bilingual translator for the purpose of publication. A bilingual translator was asked to confirm the quality of translation to maintain meanings and concepts.

Content analysis method was used to analysis the data. Content analysis is used in evaluating the data from qualitative case studies designs [13]. Data was analyzed according to method suggested by Graneheim and Lundman (2004) [16]. All of the interviews conducted by principal investigator (HS). Then, two researchers (HS, and MSK) transcribed and analyzed the data independently. Then, the meaning units as the phrases related to the participants’ experiences were identified. In the next procedure, the data were summarized as condensed meaning units, labeled with codes, and sorted into categories and sub-categories based on their similarities and differences. Finally, the same categories were abstracted and labeled with the related themes. The last two steps of the analysis process were conducted by all authors. When the codes, or categories were different by two researchers, we asked to other authors to help and data analysis. Reliability was checked using the following formula suggested by Miles and Huberman [17].

$$ \mathrm{Reliability}=\mathrm{Number}\ \mathrm{of}\ \mathrm{agreements}/\mathrm{Number}\ \mathrm{of}\ \mathrm{agreements}+\mathrm{Disagreements} $$

As a rule, the minimum percentage to demonstrate adequate levels of agreement is 75% [18]. In this study the total agreement of themes, categories, and sub-categories was 94% which shows well agreement between authors.

### Data trustworthiness

The strategies recommended by Guba [19] were used for evaluating the trustworthiness of the data. Thus, four criteria including credibility, dependability, confirmability, and transferability are necessary for confirming trustworthiness. Prolonged engagement, triangulation, member checks, peer debriefing and negative case analysis were used to ensure the credibility of the data [20]. Regarding the prolonged engagement, the researcher was actively engaged in the disaster field for 2 months while making observations and compiling field notes continually. As for triangulation, multiple researchers were to evaluate the study, which brings different perceptions of the inquiry and helps to strengthen the integrity of the findings. Regarding data triangulation or informant’s triangulation, different sources of data or instruments such as interviews, and field observation were utilized to enhance the quality of the data from different sources. In order to check the members, the analyzed and interpreted data were sent back to the participants to evaluate the interpretation and change the interpreted data if the participants were unsatisfied. In addition, peer debriefing was implemented to provide inquirers with the opportunity to test their growing insights and expose themselves to the searching questions. Finally, negative case analysis was used when the data collected from the inquiry was inconsistent with the researcher’s expectations, leading to an improvement in the credibility of the study. The data dependability was assessed by an audit trail, stepwise replication, code-recode strategy, and peer examination. Reflexive journal or practice, along with the integrity of research findings was used to assess the confirmability of data. In this regard, the researcher’s background and interest on the subject and maintaining the documents of study were used for checking the confirmability of data [20]. Further, a thick description of data, along with theoretical/purposive sampling was utilized to ensure the transferability of data [20]. The context of the interviews, codes, and extracted categories and themes were reviewed by the research team and other professional colleagues in the field of qualitative research. Using sampling with a maximum variation, the researchers were able to collect a wide variety of different comments, observations, and interpretations.

## Results

The mean age of the participants was 31, most of them were male, and had an academic education level (Table 1). First, the transcribed data from 21 interviews were analyzed, along with field note observation. The number of initial codes obtained from the interviews was 1053, which were placed in 19 sub-categories, 9 categories, and 2 themes (Table 2). Managerial and structural barriers were obtained as main themes. The categories included education, command and coordination, communication and information, rules, security, traffic and overcrowding, assessment, providing system, cultural setting.

**Table 1:** Demographic information of the participants

Variables	Number (%)
**Affected people**	**5 (23.80%)**
Participants	
**Aid workers**	**4 (19.04%)**
**Volunteers**	**6 (28.58%)**
**Humanitarian aid manager**	**6 (28.58%)**
**25–35**	**8 (38.1%)**
Age	
**36–45**	**7 (33.33%)**
**46–55**	**6 (28.57%)**
Sex	
**Male**	**14 (66.66%)**
**Female**	**7 (33.33%)**
Education Level	
**High School degree**	**3 (14.28%**
**Bachelor’s degree**	**9 (42.86%)**
**Master’s degree**	**5 (23.80%)**
**Ph.D. degree**	**4 (19.05%)**

**Table 2:** Themes, categories, sub-categories, and codes related to the challenges and barriers of humanitarian aid management in 2017 Kermanshah Earthquake

Themes	Categories	Sub-categories	Example of codes
**Managerial barriers**	**Education**	**Public education**	Lack of education of humanitarian aids in the schools
			Lack of education for managing the affected society
			Lack of familiarity with the involved organizations in humanitarian aids
		**Organizational education**	Inappropriate education and management of the enthusiastic persons
			Insufficient knowledge about the ways of assisting
			Inappropriate education and management of the local trustees
			Lack of enough education of the volunteers
	**Command and Coordination**	**Incident Command System**	Several decision-making centers
			Lack of an official administrator for disaster management
			Lack of unified incident command system
		**Coordination and Cooperation**	No coordination among the assisting organizations
			Lack of an organization for coordinating the aids and donations
			Interference of the duties of responsible organizations
	**Communication and Information**	**Social media**	Weakness of the media in managing the rumors
			Ineffective social media
			Multiplicity of sources of information and news
		**Organizational communication**	Weakness of the inter-organizational communication
			Weakness of the intra-organizational communication
	**Rules**	**Rules and Regulations**	Lack of participation rules for the celebrities
			Lack of rules and regulations for collecting cash and non-cash donations
			Lack of specified protocols
		**Supervision and Transparency**	Weakness of the participation rules for organizations
			Weakness of participation rules for the communities
			Insufficient transparency of the aids gathered by GOs and NGOs
			Insufficient supervision on the celebrities’ accounts
			Multiplicity of the governmental and non-governmental institutions for collecting donations
**Structural barriers**	**Security**	**The security of affected area**	The existence of thieves in the affected area
			Lack of a proper plan for recognizing affected people from abusing persons
		**The security of intercity area**	Insufficient security of trucks carrying the donations in the intercity routes
			Insufficient security of donors and volunteers in the intercity routes
	**Traffic and Overcrowding**	**Overcrowding of affected area**	People overcrowding
			Volunteer overcrowding
		**Traffic of the intercity area**	Congestion of vehicles
			Inappropriate management of intercity traffic
			Heavy traffic
	**Assessment**	**Need assessment**	Lack of assessing the needs of the affected area
			Inadequate consideration of sanitary needs, especially for women, kids, and elders
			Lack of using local capacities in identifying the needs
		**Vulnarability and Capacity assessment**	Lack of assessing the valnurability and capacity of the affected area
			Lack of using local people in identifying the vulnerabilities and capacities
	**Providing system**	**Distribution**	Remoteness and impracticability of some rural areas
			Insufficient distribution of the aids to the villages and distant regions
			Inequality in distributing the aids
			Delay in sending the vital aids
		**Preservation**	Ambushing the food under sunlight
			Lack of enough equipment for saving
			Peoples’ interferences in the preservation process
	**Cultural setting**	**Community trust**	People’s low trust in the governmental institutions
			People’s excessive trust in the celebrities
		**Community discipline**	People’s rush to the distribution places
			The poor culture of waiting in the queues
		**Benefactor and charity culture**	Tendency to work in the health section more than another sections
			Distrust of the benefactors in the governmental institutions
			Parallel services of the charity organizations

### Managerial barriers

#### Education

Most of the participants claimed that the lack of public and organizational education is considered as the most important barriers. Such an education encounters with weaknesses and challenges in many aspects since people are not familiar with the ways for helping and people who are interested in humanitarian aids are not informed of which kind of donation, to whom, in which time, and how should be delivered. In addition, the volunteers and local trustees are not well familiar with the ways of participation in identifying the needs and the needy people, as well as delivering and distributing the donations. Thus, not only the people affected by disasters are suffering from lack of knowledge, but also the volunteers, managers, and the relief staff and GOs are not sufficiently knowledgeable in this regard.

*“Our donors do not know which goods, when, and to which organizations should be delivered. Most of them go to the affected areas individually to provide aids. At the time of distributing the aids to the rural people, we did not know to whom we should deliver the aids. The villages’ trustees were mostly old and were not capable of receiving and distributing the donations.”*

#### Command and coordination

According to the participants, the multiplicity of the organizations responsible for the disasters is considered as a serious challenge for managing humanitarian aids and donations. The participants believed that people are confused due to the lack of information about the way they should deliver the aids. Further, the victims were not informed of where to refer for receiving the aids. Lack of a unique commandership led to the multiplicity of decision-taking centers, useless attempts, and lack of efficient and effective accountability.

*“Many organizations from governmental ones to NGOs, offices, universities, local councils collected the aids and set out for the affected districts. Some of the people received help from several organizations or institutions while some other could not get any help.”*

According to the subsequent interviews and the obtained data, assisting organizations were not sufficiently coordinated and cooperative in various levels from the management and strategic to operational levels. In other words, due to the lack of coordination, resources, staff, and equipment were sent to the affected regions but they were not coordinated.

“*The military forces, the NGOs like Red Crescent, and GOs all were simultaneously involved in the aid and rescue operations without any coordination among them. Crowding and congesting the forces and equipment were themselves transformed into a crisis.”*

#### Communications and information

These barriers were the main concerns of the participants. They were regarded rumor-fabricating and rumor-mongering as a disturbing factor in preparedness and proper response. They were dissatisfied with the weak performance of the media and lack of control on several social networks. The multiplicity of media and lack of a unique and influential management for organizing supervision and monitoring on the news, and the existence of ample inauthentic news sources were considered as the most significant challenges.

“*Different news was heard in any moment. The news broadcasted from governmental and cyber space channels were completely contradicting. There was no unique news about the casualties, damaged areas, and amount and ways of delivering aids. Many rumors were heard from people and different institutions in the cyber space.”*

#### Rules

Most of the participants regarded the lack of rules and consistent supervising system as important factors. They assumed the lack of control on the performance of celebrities and renowned persons as a significant factor for misusing this group from people’s participation and the appropriation of the gathered humanitarian monetary resources to their own benefits. In such condition, there is no supervision by any accountable organization, governmental organizations, and NGOs, and the celebrities introduce multiple bank account numbers in order to collect people’s cash as in-cash donations. Thus, many cases of misusing the aids were reported.

“*Some of the famous cinema actors gave their bank account numbers in the cyber networks to collect people’s cash aids but except for a few, most did not spend the money for the afflicted people. In addition, the supervising organizations did not prosecute the issue. Several account numbers were presented incessantly for receiving the donations from individuals and different organizations”.*

### Structural barriers

#### Security

The participants reported that lack of adequate security is the most significant barrier for delivering humanitarian donations and aids. They asserted that the security of the mobilized facilities and equipment, especially the ones sent overland, were not duly provided. Additionally, the thieves’ rush from other regions to the affected areas, overcrowding, and fraud all with malicious intentions created some disorder in security.

“*We heard that the trucks having been carrying the same aids were assaulted and the aids were stolen. Perhaps, they were actually in need and did not intend to steal anything, but in my idea, it would rather have been better to escort the trucks by security forces”.*

#### Assessment

Lack of assessing the needs and capacity of the area is influenced by two main challenges reported by the participants. The local trustees and individuals believed that their opinions were not respected in identifying the need. In addition, the responsible organizations distributed the aids without considering the real necessities of people. According to the participants, having no plan for needs and capacity assessment can result in wasting the resources, along with injustice in the distribution. Further, the needs of vulnerable groups, especially the elders, children, disabled, and families with poor custodian or no custodian were disregarded.

*“As a rescuer who was the supervisor of an assisting group, I did not know which village or district of the cities was more in need and whether any aid had been sent to those regions or not. Some other people came to receive goods, foods, clothes, and other things. We had no idea which families had disabled members or elders or pregnant women, and who had lost their husbands because they were not able to refer to assisting centers to receive aids”.*

#### Providing system

According to the interviews, preserving and distributing the humanitarian aids are considered as the barriers and challenges regarding providing system, which can be divided into three main phases. The first phase was the barriers related to the collection and arrival of the aids and donations to the affected areas. In fact, there was no procrastination in common participation in delivering the aids and the inaccessibility of the damaged region due to the damaged connecting roads, and the remoteness of some villages led to the prediction in transferring the aids to the regions. The second phase was the challenge of preserving the goods in the affected areas. The participants reported that foods were preserved under sunlight and rain in an unhealthy condition, and some mercenary persons rushed to the deposit places and stole things many times. The third phase involved the challenges related to the distributions such as having no preceding plan for distribution, non-observation of equivalence in distribution, and untargeted distribution.

“*The aids were arrived to some remote villages with delay. In other villages whose roads were damaged, the food packages were thrown down from rescue helicopters which caused the packages to become spoiled due to crashing into the ground. People’s aids were collected in one of the city squares and they were covered by a canvas. The foods on the ground were in contact with the rain water and they were exposed to sunlight”.*

#### Cultural setting

The participants believed that cultural setting plays a significant role in managing the humanitarian aids. They emphasized that lack of community trust in GOs versus the excessive trust they had in celebrities and renowned persons as one of the main challenges. The responsible GOs and the NGOs were inattentive to people’s culture and customs, leading to some problems in managing humanitarian aids. Furthermore, people’s inattentiveness for waiting in the queues was regarded as another cultural barrier*.*

“*If people had trusted the GOs as they trusted the celebrities, there would not have been that much misuse. People are also to be blamed because they are affected by emotions for collecting aids in the first days after disasters. In addition, the afflicted people rush for receiving the aids. For instance, when a truck arrived for assisting, they did not stand in queues but they wanted to get the goods all at once”.*

The participants emphasized that the benefactors had remarkable and unbelievable participation during rescuing, relieving, and rehabilitation. They believed that the benefactors had not adequate knowledge in this regard. Further, the benefactors have a tendency for humanitarian actions in health services rather than other domains and they scarcely cooperate with governmental organizations.

“*There are many benefactors in Iran but unfortunately they lack adequate awareness and knowledge and they do not act untidily. It would be better if benefactors become coordinated with each other and each supplies some parts of the requirements for the injured people. The benefactors visited the affected areas carrying goods on their own. I wish a unique organization can carry out such things”.*

## Discussion

Lack of appropriate education for humanitarian aids during disasters is considered as one of the main challenges. People, managers, relief staff, and benefactors were devoid of the adequate knowledge on humanitarian aids. Since different organizations with different potentialities, capacities, duties, and interests participate in the humanitarian assistance, it is necessary to focus on improving the performances, designing the standards and educations, and promoting the educational courses by means of sharing their experience in the stage of preparedness. Some studies suggested an attempt for reducing unnecessary aids with low priority through public and professional education [10].

The existence of different broadcasting, rumor-fabricating, and rumor-mongering sources is considered as one of the challenges in the study due to the lack of education and regulation of the news sources and their control. In addition, the reliability of the information and confronting with rumors are regarded as the main concerns in the response phase of disasters because the social networks can present inauthentic information, rumors, and fictitious news due to the lack of any control process and the low quality of the information [21, 22]. Therefore, adequate education to the media added to a governmental supervision seems crucial.

Further, communication barriers in delivering the humanitarian aids were emphasized by some participants. Todays, the social networks are too comprehensive and play a significant role in creating communication and coordination among the assisting organizations due to their fast broadcasting of information. The NGOs and volunteers can use social networks for collecting donations in the least time possible [23].

Furthermore, collecting exact, authentic, and effective information is considered as one of the key factors in the process of distributing humanitarian aids in disasters. Thus, attention should be paid on the unreal and inauthentic reports, which can negatively affect the participation process in delivering and distributing the humanitarian helps [24]. Managing humanitarian aids during disasters is naturally difficult due to the variety of the beneficiaries. In such cases, receiving and sharing information about the infrastructures and supplying the required resources are significant for effective response and social networks can get information about the number of people afflicted by the disasters through internet and make it accessible in order to have a fast and effective response [25, 26].

According to some participants, the needs of injured people are different during a disaster. Donations should be consistent with the receivers’ requirements rather than the donators’ preferences [27]. Mobilizing the aids without need assessment leads to the accumulation of abundant unnecessary sources in the affected areas. Additionally, ignoring the requirements of vulnerable groups such as women, children, elders, and the disabled at the time of disasters are considered as another barrier for managing aids and donations during the disasters. These groups may not be able to supply their needs due to cultural, religious, and other personal limitations [28, 29].

Some participants maintained that lack of donors’ trust in governmental institutions is one of the main barriers for managing donations, which causes donations to be collected through unofficial individuals like celebrities which can result in misusing what has been provided [2]. Another structural impediment regarding donations in disasters is related to lack of any respect for cultural beliefs and values of the affected people. In this regard, donations will not be utilized if they are not congruent with the culture of that community after sending to the affected areas [30].

Lack of a systematic plan for food preservation and food security was considered as another challenge introduced by the participants. The significance of food security and feeding was emphasized in Sendai framework and sustainable development goals [31]. Lack of concern for food variety in the mobilized aids is regarded as another challenge proposed by the participants. It is necessary to guarantee the variation of foods and consumption of nourishing favorite nutrients, particularly in the first days after disasters [32]. Inequality and injustice in distribution and rushing for distributing centers are considered as some challenges regarding distributing the aids. Regarding the inevitability of identification and registration of the beneficiaries, it seems necessary for participants to adopt a controlled and fair strategy for distribution [33]. The participants stated that depositories were defected and the food was exposed to sunlight and insects for a long. Food and medicine are the first priorities in planning for maximizing security in disasters and they should be accommodated in a dry and cool place with air-conditioning systems [34].

In addition, some addressed that coordination among humanitarian organizations is a great challenge in managing donations. Lack of a managing commitment, improper organizational structure for sharing knowledge, as well as lack of a specified policy and planning are considered as the main barriers for coordination [35]. Further, establishing cluster approach and network subgroups in the affected region can lead to the contribution of coordination and collaboration in managing humanitarian donations [36].

Since the government plays a fundamental role in organizing and coordinating the humanitarian assistance operations [37], authorization generates a potential ability for responding to the disasters based on law and constitution [38]. Integrated management of the processes, policies, and legislations can play a fundamental role in managing the aids among people [39]. Additionally, organizing and coordinating the effective participation of government and benefactors can increase the opportunities in responding to disasters and casualties [40].

In addition, providing security in the affected areas is considered as one of the main problems reported by some participants [41, 42]. Further, insecurity brings challenges for humanitarian organizations and puts the distributors of aids and people at risk [43]. Distributing aids through adopting a consistent management of provisional duties such as depository keeping, qualified transfer system, and fair access to aids can be effective in managing donations and preventing forage by thieves [43].

### Limitations

The present study clearly had some limitations. One potential limitation of the current study was that this study conducted in the Iranian context that it can be limited in generalizability to other countries. However, it seems that given the shared challenges, the results can be applicable in the same countries with same setting. The language was a barrier in the affected area of Kermanshah in which Kurdish is a native language. Although most of the interviewees speak in Persian, some of the affected people could speak Kurdish. To overcome the limitation, HS, DP, and AS, as three native colleagues, helped the researchers to translate Kurdish words into Persian.

## Conclusion

Humanitarian aid management during disasters has many challenges. Adopting an efficient management and appropriate policies for humanitarian aids and donations, as well as eliminating structural and policy barriers can improve the performance of management. Further, developing public education and evaluating the needs based on cultural factors, as well as supervising and clarifying the aids can help reduce the challenges and barriers. In addition, it is essential to establish a lead agency with sufficient authority to supervise the collection, transport and distribute humanitarian aids and donor managements. Since involving a variety of organizations and individuals, especially the celebrities, in collecting and distributing such aids can lead to some sources of misuse and sometimes abuse, it is recommended that the government should provide adequate training and preparation for their intervention and supervision by adopting the related laws. The registry system can help manage this problem. In addition, vulnerable groups such as the elderly, children, homeless people, disabled individuals, and pregnant women should be considered while distributing the aids. Finally, training local authorities and trustees should be highlighted in the preparedness plans. Furthermore, these findings as a scientific document, will be available to managers of NGOs and GOs involved in rescue and relief and humanitarian aid management for use in the field. Moreover, the knowledge created by this study, can lead to awareness of policy makers and managers in other countries about the challenges of humanitarian aid management and help them to better preparedness and respond against future disasters.

## Data Availability

The datasets used during the current study are available from the corresponding author upon reasonable request.

## Abbreviations

NGO: Non-governmental Organization
GO: Governmental Organization
SDGs: Sustainable Development Goals

## References

[1] Jahangiri K, Izadkhah YO, Jamaledin TS (2011). A comparative study on community-based disaster management in selected countries and designing a model for Iran. Disaster Prev Manage Int J.

[2] Safarpour H. Donors Management in Disasters: Kermanshah Earthquake Experience, Iran Red Crescent Med J. 2018;20(11):e84942.

[3] Chang Y, Wilkinson S, Potangaroa R, Seville E (2011). Donor-driven resource procurement for post-disaster reconstruction: constraints and actions. Habitat Int.

[4] Ferrer JM, Martín-Campo FJ, Ortuño MT, Pedraza-Martínez AJ, Tirado G, Vitoriano B (2018). Multi-criteria optimization for last mile distribution of disaster relief aid: test cases and applications. Eur J Oper Res.

[5] Andrighetto L, Baldissarri C, Lattanzio S, Loughnan S, Volpato C (2014). Human-itarian aid? Two forms of dehumanization and willingness to help after natural disasters. Br J Soc Psychol.

[6] Heaslip G, Kovács G, Haavisto I (2018). Cash-based response in relief: the impact for humanitarian logistics. J Hum Logistics Supply Chain Manage.

[7] Kovács G, editor. Where next? The future of humanitarian logistics. In: Humanitarian Logistics Meeting the Challenge of Preparing for and Responding to Disasters. Kogan Page. London. 2014. pp. 275–86. http://www98.griffith.edu.au/dspace/handle/10072/39729.

[8] Mohd S, Fathi M, Harun A. Humanitarian Aid Distribution Framework for Natural Disaster Management. Int Arch Photogrammetry Remote Sensing Spatial Inform Sci. 2018;42(3/W4).

[9] Ismail D, Majid TA, Roosli R, Ab SN (2014). Project management success for post-disaster reconstruction projects: international NGOs perspectives. Procedia Economics and Finance.

[10] Varella L, Gonçalves M, editors. Collaboration: A critical success factor in the logistics of Donations Management. 27th Conference-Production and Operations Management Society; 2016.

[11] Patel R, Wild H. To do no harm: humanitarian aid in conflict demands political engagement. Disaster Med Public Health Preparedness. 2018:1–2. 10.1017/dmp.2017.133 29350611

[12] Peyravi M, Marzaleh MA, Khorram-Manesh A (2019). An overview of the strengths and challenges related to health on the first 10 days after the large earthquake in the west of Iran, 2017. Iran J Public Health.

[13] Nieswiadomy RM. Foundation of Nursing Research. Sixth Edition. London: Pearson; 2011. pp. 352.

[14] Hsieh H-F, Shannon SE (2005). Three approaches to qualitative content analysis. Qual Health Res.

[15] Elo S, Kääriäinen M, Kanste O, Pölkki T, Utriainen K, Kyngäs H (2014). Qualitative content analysis: a focus on trustworthiness. SAGE Open.

[16] Graneheim UH, Lundman B (2004). Qualitative content analysis in nursing research: concepts, procedures and measures to achieve trustworthiness. Nurse Educ Today.

[17] Miles MB, Huberman AM, Huberman MA, Huberman M. Qualitative data analysis: An expanded sourcebook. Thousand Oaks: SAGE Publications, Inc; 2nd edition. 1994. pp. 352.

[18] Cook KE (2012). Reliability assessments in qualitative health promotion research. Health Promot Int.

[19] Guba EG (1981). Criteria for assessing the trustworthiness of naturalistic inquiries. Ectj..

[20] Anney VN (2014). Ensuring the quality of the findings of qualitative research: looking at trustworthiness criteria. J Emerg Trends Educ Res Policy Studies (JETERAPS).

[21] Marbouti M, Maurer F. Social Media Use During Emergency Response – Insights from Emergency Professionals. 15th Conference on e-Business, e-Services and e-Society (I3E). Swansea, United Kingdom: Springer International Publishing; 2016. pp.557–66. https://hal.inria.fr/hal-01702151/file/396007_1_En_49_Chapter.pdf.

[22] Wang B. Crisis communication on social media: behaviors and patterns. New York: State University of New York at Buffalo; 2019. http://hdl.handle.net/10477/79307.

[23] Mauroner O, Heudorfer A (2016). Social media in disaster management: how social media impact the work of volunteer groups and aid organisations in disaster preparation and response. Int J Emerg Manag.

[24] Mohd S, Fathi MS, Harun AN. Information management for humanitarian aid distribution system in Malaysia. In IOP Conference Series: Materials Science and Engineering: IOP Publishing; 2019;513(1):012012.

[25] Snook KR, Duke CH, Finch KC, Fu K-W, Tse ZTH, Adhikari A (2015). How social media is used during natural disasters, environmental disasters, and other environmental concerns: a scoping review.

[26] Yoo E, Rand W, Eftekhar M, Rabinovich E (2016). Evaluating information diffusion speed and its determinants in social media networks during humanitarian crises. J Oper Manag.

[27] Nelson T (2012). Determinants of disaster aid: donor interest or recipient need? Global change. Peace & Security.

[28] Daddoust L, Khankeh H, Ebadi A, Sahaf R, Nakhaei M, Asgary A (2018). The vulnerability of the Iranian elderly in disasters: qualitative content analysis. Iran J Nurs Midwifery Res.

[29] Sohrabizadeh S, Jahangiri K, Jazani RK, Babaie J, Moradian MJ, Rastegarfar B. Women’s Challenges and Capabilities in Disasters: a Case Report of the Twin Earthquakes of Eastern Azerbaijan, Iran. PLOS Currents Disasters. 2017 Feb 22 . Edition 1. 10.1371/currents.dis.2cff3d6e9e0c3a597f873bf29e712370. PMC5325668 28286700

[30] Wang Y, Tang Y-Y, Wang J (2015). Cultural Differences in Donation Decision-Making. PloS one.

[31] Abarca-Gómez L, Abdeen ZA, Hamid ZA, Abu-Rmeileh NM, Acosta-Cazares B, Acuin C (2017). Worldwide trends in body-mass index, underweight, overweight, and obesity from 1975 to 2016: a pooled analysis of 2416 population-based measurement studies in 128·9 million children, adolescents, and adults. Lancet.

[32] De Pee S, Taren D, Bloem MW. Nutrition and health in a developing world: Humana Press; Springer; 3rd edition; 2017. pp. 827. https://link.springer.com/book/10.1007%2F978-3-319-43739-2.

[33] Nozhati S, Sarkale Y, Ellingwood BR, Chong EK, Mahmoud H. A modified approximate dynamic programming algorithm for community-level food security following disasters. arXiv preprint arXiv:180400250. 2018.

[34] Nozue M, Ishikawa-Takata K, Sarukura N, Sako K, Tsuboyama-Kasaoka N (2014). Stockpiles and food availability in feeding facilities after the great East Japan earthquake. Asia Pac J Clin Nutr.

[35] Kabra G, Ramesh A, Arshinder K (2015). Identification and prioritization of coordination barriers in humanitarian supply chain management. Int J Disaster Risk Reduction.

[36] Handayani NU, Mustikasari A. Coordination and Collaboration Functions of Disaster Management Centers for Humanitarian Logistics: a Case Study at Merapi Eruption. MATEC Web of Conferences. 2018;159:01046.

[37] Hesama L (2019). Review of the commitment of governments to accept and send humanitarian aid in natural disasters. J Public Law Stud Tehran Univ.

[38] Rumbach A (2016). Decentralization and small cities: towards more effective urban disaster governance?. Habitat Int.

[39] Elbert R, Gerdes U-T, Kaiser G, Sarnow T (2018). Governance and moderating effects of environmental uncertainty: the impact on performance in horizontal logistics cooperations. Logist Res.

[40] Pirani D, Yousefi K, Sahebi A (2019). Disasters; the opportunity of reconstruction and development: the successful experience of the 2017 Kermanshah earthquake. Iran Red Crescent Med J.

[41] Roth S (2015). Aid work as edgework–voluntary risk-taking and security in humanitarian assistance, development and human rights work. J Risk Res.

[42] Ghodsi H, Khani Jazani R, Sohrabizadeh S, Kavousi A (2019). The resiliency of humanitarian aid Workers in Disasters: a qualitative study in the Iranian context. Iran Red Crescent Med J.

[43] Schneiker A. Humanitarian NGOs,(in) security and identity: Epistemic communities and security governance. 1st Edition ed. London: Routledge; 2016. p. 164. 10.4324/9781315587608.

